# Identification of a Phenylthiazole Small Molecule with Dual Antifungal and Antibiofilm Activity Against *Candida albicans* and *Candida auris*

**DOI:** 10.1038/s41598-019-55379-1

**Published:** 2019-12-12

**Authors:** Haroon Mohammad, Hassan E. Eldesouky, Tony Hazbun, Abdelrahman S. Mayhoub, Mohamed N. Seleem

**Affiliations:** 1 Department of Comparative Pathobiology, College of Veterinary Medicine, Purdue University, 625 Harrison St., West Lafayette, IN 47907 USA; 2 Bindley Bioscience Center, Purdue University, 1201 W State St., West Lafayette, IN 47907 USA; 3 Department of Medicinal Chemistry and Molecular Pharmacology, College of Pharmacy, Purdue University, 575 Stadium Mall Dr., West Lafayette, IN 47907 USA; 4 Department of Pharmaceutical Organic Chemistry, College of Pharmacy, Al-Azhar University, Cairo, 11884 Egypt; 5 University of Science and Technology, Nanoscience Program, Zewail City of Science and Technology, October Gardens, 6th of October, Giza, 12578 Egypt; 6 Purdue Institute of Inflammation, Immunology, and Infectious Disease, 610 Purdue Mall, West Lafayette, IN 47907 USA

**Keywords:** Phenotypic screening, Fungal infection, Biofilms, Preclinical research

## Abstract

*Candida* species are a leading source of healthcare infections globally. The limited number of antifungal drugs combined with the isolation of *Candida* species, namely *C*. *albicans* and *C*. *auris*, exhibiting resistance to current antifungals necessitates the development of new therapeutics. The present study tested 85 synthetic phenylthiazole small molecules for antifungal activity against drug-resistant *C*. *albicans*. Compound **1** emerged as the most potent molecule, inhibiting growth of *C*. *albicans* and *C*. *auris* strains at concentrations ranging from 0.25–2 µg/mL. Additionally, compound **1** inhibited growth of other clinically-relevant yeast (*Cryptococcus*) and molds (*Aspergillus*) at a concentration as low as 0.50 µg/mL. Compound **1** exhibited rapid fungicidal activity, reducing the burden of *C*. *albicans* and *C*. *auris* below the limit of detection within 30 minutes. Compound **1** exhibited potent antibiofilm activity, similar to amphotericin B, reducing the metabolic activity of adherent *C*. *albicans* and *C*. *auris* biofilms by more than 66% and 50%, respectively. Furthermore, compound **1** prolonged survival of *Caenorhabditis elegans* infected with strains of *C*. *albicans* and *C*. *auris*, relative to the untreated control. The present study highlights phenylthiazole small molecules, such as compound **1**, warrant further investigation as novel antifungal agents for drug-resistant *Candida* infections.

## Introduction

Fungi are ubiquitous in nature with most species being innocuous to humans. However, species of *Candida* are responsible for the majority of fungal infections reported in the healthcare setting globally. *Candida* infections range from superficial skin lesions to oral thrush, vulvovaginal candidiasis, and bloodstream infections (BSIs). In hospital intensive care units (ICUs), species of *Candida* are the third leading source of infection, responsible for almost one-fifth of all ICU infections^1^. Within the general pediatric population, nearly 9% of all nosocomial infections in Europe and the United States are due to *Candida* species^2,3^. More specifically, *Candida* species are the fourth-leading source of BSIs in the general population^4^. Within the pediatric population, candidemia is the third-leading cause of BSIs^2,3^. Disseminated candidiasis has mortality rates reportedly ranging from 40–60% and results in nearly $2 billion in health care costs annually in the U.S. alone^5,6^.

*C*. *albicans*, commensal of the gastrointestinal (GI) and reproductive tracts, oropharyngeal cavity, and skin of humans is the predominant species of *Candida* linked to opportunistic infections and remains the leading source of candidemia in ICUs worldwide^4,7^. However, the incidence of infections due to non-albicans species, including *C*. *glabrata*, *C*. *krusei*, and *C*. *parapsilosis*, has increased globally particularly in Australia, the United States, and in Europe^4^. Treatment of *Candida* infections is challenging for clinicians as only three antifungal drug classes are available – azoles, polyenes, and echinocandins. Though azoles, such as fluconazole, were the mainstay treatment option for *Candida* infections for nearly two decades, a decrease in susceptibility to azoles by *Candida* species has been reported^5,8^. Thus, recent guidelines in the U.S. and Europe have recommended the use of echinocandins as frontline treatment options for *Candida* infections, and fluconazole for step-down therapy, particularly for invasive infections in immunocompromised patients^5,9,10^. However, increased use of echinocandins has resulted in more strains of *C*. *albicans*, *C*. *glabrata*, and *C*. *parapsilosis* exhibiting reduced susceptibility to echinocandins^4^. The emergence of the highly drug-resistant *Candida auris*, first identified in 2009 from a patient in Japan, has compounded the treatment challenge associated with *Candida* infections. The U.S. Centers for Disease Control and Prevention (CDC) has reported that nearly 90% of *C*. *auris* isolates exhibit resistance to fluconazole, nearly 30% resistance to amphotericin B, and less than 5% resistance to echinocandins^11^. The problem is worse in England where nearly all *C*. *auris* isolates exhibit resistance to fluconazole, almost 20% resistance to amphotericin B, and nearly 10% resistance to echinocandins^12^. Of deep concern are *C*. *auris* isolates exhibiting resistance to drugs in all three antifungal classes^13,14^.

The limited number of antifungal drugs combined with the rise in resistance to current antifungals has resulted in the U.S. CDC categorizing drug-resistant *C*. *albicans* and *C*. *auris* as urgent public health threats for which new antifungal agents are needed^15^. This provided the impetus for us to evaluate a library of synthetic phenylthiazole compounds, developed in-house, for antifungal activity against clinically-relevant species of *Candida*. Screening identified one compound (**1**) as a promising lead compound with potent inhibitory activity against fluconazole-resistant *C*. *albicans*. The aim of this study was to investigate compound **1**’s antifungal activity against a wider panel of drug-resistant *Candida* isolates, ability to inhibit formation of and disrupt *Candida* biofilms, and to protect nematodes infected with drug-resistant, highly-virulent strains of *C*. *albicans* and *C*. *auris*.

## Results

### Initial screening of phenylthiazole compound library against fluconazole-resistant *C*. *albicans*

We initially evaluated the antifungal activity of 85 phenylthiazole compounds against *C*. *albicans* P60002, which exhibited high-level resistance to fluconazole (MIC > 64 µg/mL) (Table S1). Of the compounds screened, 55 were inactive (MIC > 64 µg/mL) while 18 compounds exhibited weak antifungal activity (MIC ranged from 16 to 64 µg/mL). Eleven compounds exhibited moderate-to-good antifungal activity with MIC values against *C*. *albicans* P60002 of 2 µg/mL (for compound **10**), 4 µg/mL (for compounds **2**, **3**, **11**, **17**, **21**, **23** and **65**), or 8 µg/mL (for compounds **13**, **19**, and **36**). Only compound **1** exhibited potent antifungal activity against *C*. *albicans* P60002 (MIC = 0.50 µg/mL), similar to the control antifungal amphotericin B. Thus compound **1** was selected as the lead compound for further investigation.

### Evaluation of compound 1’s antifungal activity against a wider panel of fungal isolates

Compound **1** was tested against a panel of fungal isolates to determine its spectrum of activity (Table 1). First, compound **1** was evaluated against five additional *C*. *albicans* strains. The compound showed potent antifungal activity (MIC = 0.50 µg/mL) against two strains of fluconazole-sensitive *C*. *albicans* (MIC of fluconazole = 0.50 µg/mL) including one strain (NR-29365) exhibiting resistance to amphotericin B (MIC = 2 µg/mL). Against two additional *C*. *albicans* strains (NR-29446 and ATCC MYA-573) exhibiting high-level resistance to fluconazole (MIC > 64 µg/mL), compound **1** was slightly less effective (MIC = 2 µg/mL). Compound **1** also showed similar activity (MIC = 2 µg/mL) against *C*. *albicans* ATCC 64124, a strain exhibiting resistance to both amphotericin B (MIC = 2 µg/mL) and fluconazole (MIC > 64 µg/mL).

**Table 1:** Antifungal activity (MIC, in µg/mL) of compound **1** against species of *Candida*, *Cryptococcus*, and *Aspergillus*.

Fungal Strain	Compound 1	Amphotericin B	Fluconazole
*Candida albicans* NR-29351	0.50	0.50	0.50^a^
*Candida albicans* NR-29365	0.50	2	0.50^a^
*C*. *albicans* NR-29446	2	0.50	>64^a^
*C*. *albicans* ATCC MYA-573	2	1	>64^a^
*C*. *albicans* ATCC 64124	2	2	>64
*Candida auris* 381	2	0.50	1
*Candida auris* 383	2	1	>64
*Candida auris* 384	2	1	>64
*Candida auris* 385	2	2	>64
*Candida auris* 386	2	2	>64
*Candida auris* 387	2	1	>64
*Candida auris* 389	2	2	>64
*Candida auris* 390	2	2	>64
*Candida glabrata* ATCC MYA-2950	1	1	32^a^
*Candida glabrata* ATCC 66032	0.25	0.50	16^a^
*Candida parapsilosis* ATCC 22019	0.50	0.50	1^a^
*Candida tropicalis* ATCC 1369	1	0.50	1^a^
*Candida tropicalis* ATCC 13803	1	0.50	0.50^a^
*Cryptococcus gattii* NR-43208	0.50	1	2^a^
*Cryptococcus gattii* NR-43209	0.50	1	8^a^
*Cryptococcus neoformans* NR-41292	0.50	1	8^a^
*Aspergillus fumigatus* NR-35302	2	2	>64
*Aspergillus fumigatus* NR-35304	2	2	>64
*Aspergillus fumigatus* NR-41312	4	2	>64

^a^MIC data previously presented in reference^16^.

The activity of compound **1** was evaluated next against non-albicans species of *Candida*, including strains of *C*. *auris*, *C*. *glabrata*, *C*. *parapsilosis*, and *C*. *tropicalis* (Table 1). Compound **1** inhibited growth of all eight strains of *C*. *auris* at a concentration of 2 µg/mL. Fluconazole was ineffective against seven of the *C*. *auris* strains (MIC > 64 µg/mL) while amphotericin B’s MIC values ranged from 0.50 to 2 µg/mL against the same strains. Against two strains of *C*. *glabrata* resistant to fluconazole, compound **1** (MIC ranged from 0.25 to 1 µg/mL) was as effective as amphotericin B (MIC ranged from 0.50 to 1 µg/mL). Against three strains of *C*. *parapsilosis* and *C*. *tropicalis*, compound **1** (MIC ranged from 0.50 to 1 µg/mL) had similar potency to both fluconazole (MIC ranged from 0.50 to 1 µg/mL) and amphotericin B (MIC = 0.50 µg/mL). MIC data for fluconazole determined against all strains of *C*. *albicans* (with the exception of *C*. *albicans* ATCC 64124), *C*. *glabrata*, *C*. *parapsilosis*, and *C*. *tropicalis* were reported in a previous study^16^.

Of the nearly 1.5 million deaths attributed to fungal infections globally each year, most fatalities are due to species of *Candida*, *Cryptococcus*, and *Aspergillus*^17^. To gauge the spectrum of antifungal activity of the phenylthiazole compounds, we evaluated compound **1** against strains of pathogenic yeast (*C*. *neoformans* and *C*. *gattii*) and molds (*A*. *fumigatus*) (Table 1). Against the three strains of *Cryptococcus* tested, compound **1** (MIC = 0.50 µg/mL) was more potent than amphotericin B (MIC = 1 µg/mL) or fluconazole (MIC ranged from 2 to 8 µg/mL). MIC data for fluconazole against all strains of *Cryptococcus* were reported in a previous study^16^. Against three pathogenic strains of *A*. *fumigatus*, compound **1** (MIC ranged from 2 to 4 µg/mL) was as effective as amphotericin B (MIC = 2 µg/mL) while fluconazole was inactive (MIC > 64 µg/mL).

### Compound 1 has fungicidal activity against both *C*. *albicans* and *C*. *auris*

**Figure 1:**
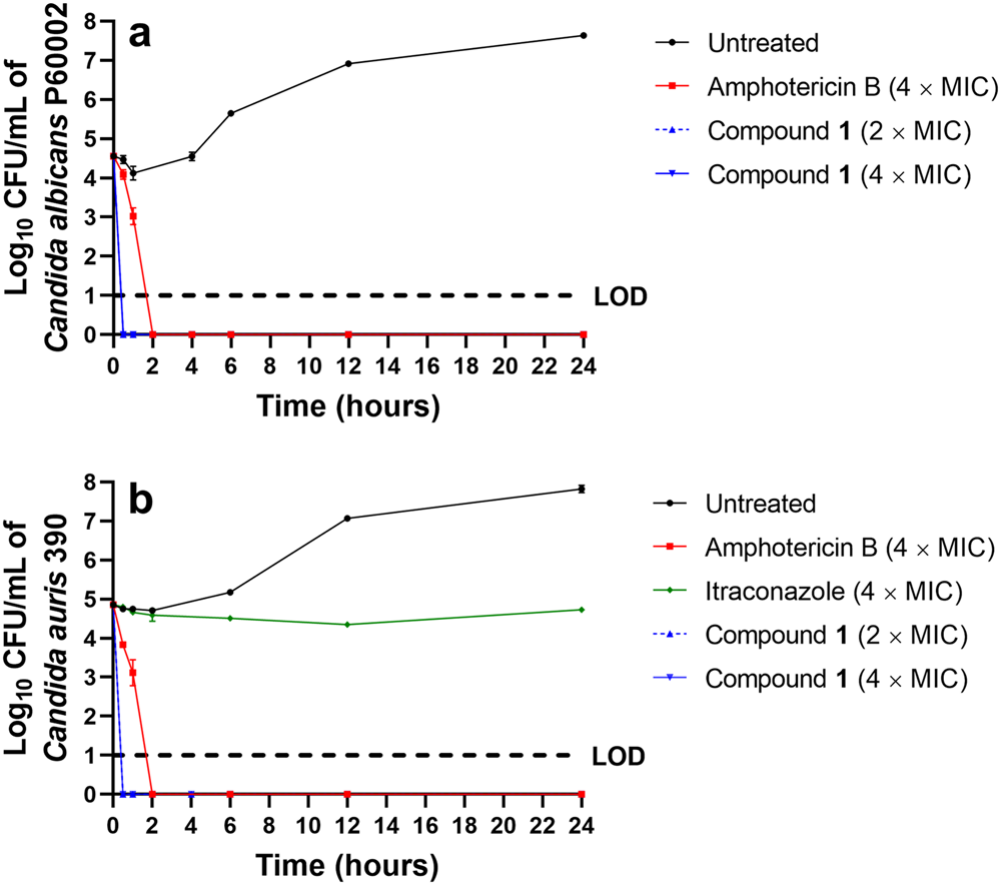
Time-kill analysis of compound 1, itraconazole, and amphotericin B. Test agents were evaluated against (**a**) *C*. *albicans* P60002 and (**b**) *Candida auris* strain 390 over a 24-hour incubation period at 35 °C. DMSO served as a negative control. Error bars represent standard deviation values. Dashed horizontal lines (---) represents the limit of detection (LOD) for the assay.

To address whether compound **1** exhibits fungistatic or fungicidal activity, a time-kill assay was conducted. Against *C*. *albicans* P60002, compound **1** (at both 2 × MIC and 4 × MIC) exhibited rapid fungicidal activity, reducing fungal viability below the limit of detection (10 CFU/mL) within 30 minutes (Fig. 1a). This was similar to the fungicidal effect observed with amphotericin B^18,19^. No fungal regrowth was observed in the presence of either compound **1** or amphotericin B during the assay (24 hours), indicating the absence of resistant mutants. A similar result was obtained when compound **1** and amphotericin B were evaluated against *C*. *auris* strain 390. Compound **1** and amphotericin B were both fungicidal with *C*. *auris* viability decreasing below the limit of detection (10 CFU/mL) within 30 minutes and two hours, respectively (Fig. 1b). Itraconazole (at 4 × MIC) exhibited bacteriostatic activity against *C*. *auris* 390. Itraconazole (MIC = 1 µg/mL) was used in place of fluconazole as *C*. *auris* 390 is not sensitive to fluconazole. We were unable to conduct a time-kill assay for azole antifungals against *C*. *albicans* P60002 as this strain is highly-resistant to azoles, including to itraconazole (MIC > 16 µg/mL).

### Investigation of compound 1’s ability to inhibit *Candida* biofilm formation

**Figure 2:**
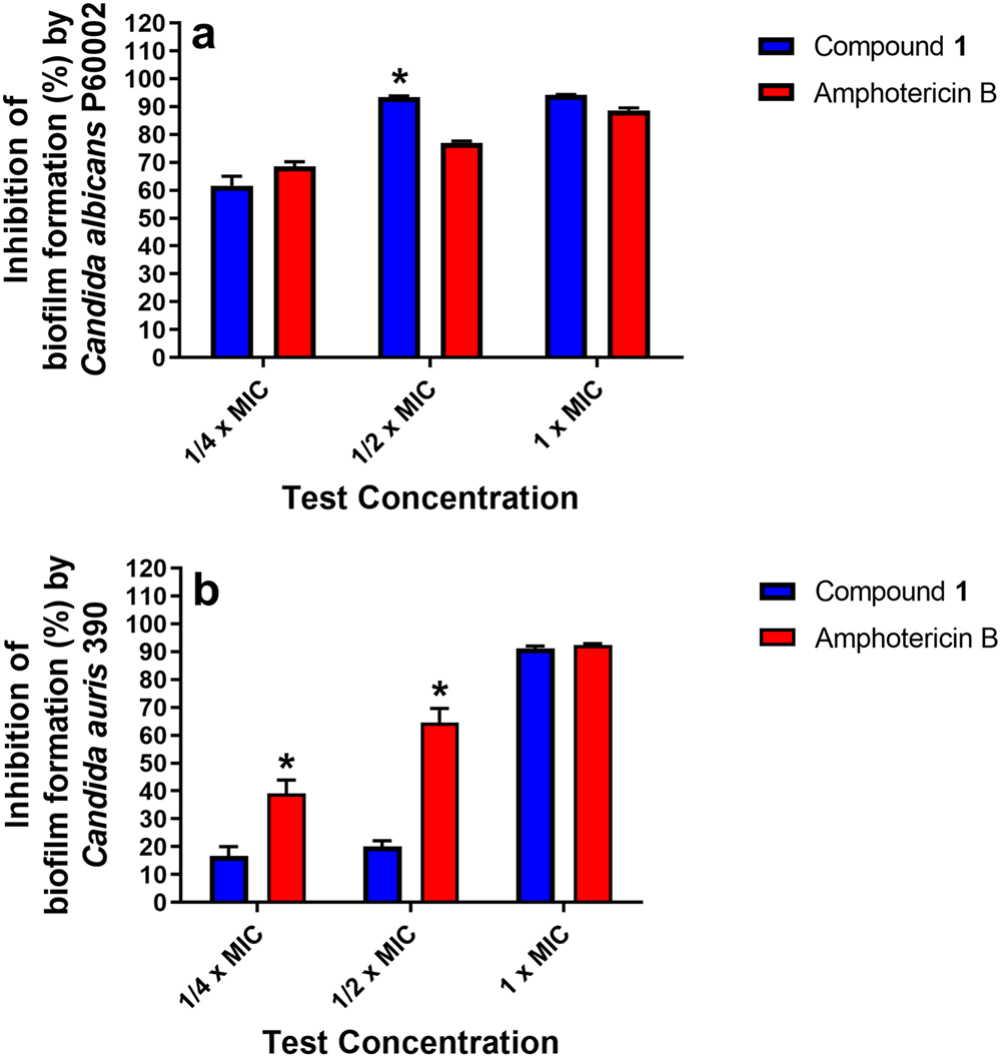
Inhibition of *Candida* biofilm formation. Compound **1** and amphotericin B were evaluated for their ability to inhibit biofilm formation by (**a**) *C*. *albicans* P60002 and (**b**) *C*. *auris* strain 390. An overnight suspension of *C*. *albicans* was diluted in RPMI-1640 medium supplemented with MOPS (to achieve a starting inoculum ~5 × 10^5^ CFU/mL) and exposed to compound **1** or amphotericin B (at the indicated concentrations) or left untreated for 24 hours at 35 °C to permit biofilm formation. The biofilm was stained with 0.1% crystal violet, de-stained with ethanol, and biofilm mass quantified at OD_595_. Data represents percent inhibition of biofilm mass by compound **1** relative to the untreated control. Data were analyzed via an unpaired one-way t-test (*P* < 0.05). Asterisks (*) indicate statistical difference between amphotericin B-treated and compound **1**-treated wells.

A major virulence factor used by *Candida* species to evade the host immune response is the formation of biofilms. The three-dimensional structure of biofilms and the induction of drug efflux pumps hinders penetration of most antifungal drugs. The formation of biofilms by *Candida* on the mucosa or endothelium in humans or on the surface of implantable medical devices has been linked to recurrent infections and treatment failure with antifungal therapy^20,21^. Indeed *Candida* biofilms present on medical devices such as catheters and orthopedic implants often are recalcitrant to treatment with antifungal drugs with the only recourse often is to remove and replace infected devices^20^. We therefore tested the ability of compound **1** to inhibit formation of biofilms by both *C*. *albicans* and *C*. *auris* using a standard microtiter dish biofilm formation assay. Compound **1** exhibited dose-dependent inhibition of *Candida* biofilm formation. Against *C*. *albicans* P60002, at ¼ × MIC, compound **1** reduced biofilm formation by 61.7% while amphotericin B reduced biofilm formation by 68.6% (Fig. 2a), relative to the untreated control wells. At ½ × MIC, compound **1** (93.4% reduction) was more effective than amphotericin B (77% reduction) at inhibiting *C*. *albicans* biofilm formation (*P* = 0.0014). Amphotericin B (88.7% reduction) and compound **1** (94.2% reduction) were equipotent at 1 × MIC in inhibiting *C*. *albicans* biofilm formation. Both compound **1** and amphotericin B were more effective at reducing biofilm formation by *C*. *albicans* than *C*. *auris*. When tested against *C*. *auris* strain 390, compound **1** was ineffective at subinhibitory concentrations as it inhibited biofilm formation only by 16.7% (at ¼ × MIC) and 20.1% (at ½ × MIC) (Fig. 2b). Amphotericin B was more effective at ¼ × MIC and ½ × MIC, as it reduced *C*. *auris* biofilm formation by 39% (*P* = 0.0439; Fig. 2b) and 64.6% (*P* = 0.0263). At 1 × MIC, both compound **1** (91.2% reduction) and amphotericin B (92.4% reduction) were equally effective at inhibiting *C*. *auris* biofilm formation (Fig. 2b).

### Compound 1 disrupts adherent *Candida* biofilms

**Figure 3:**
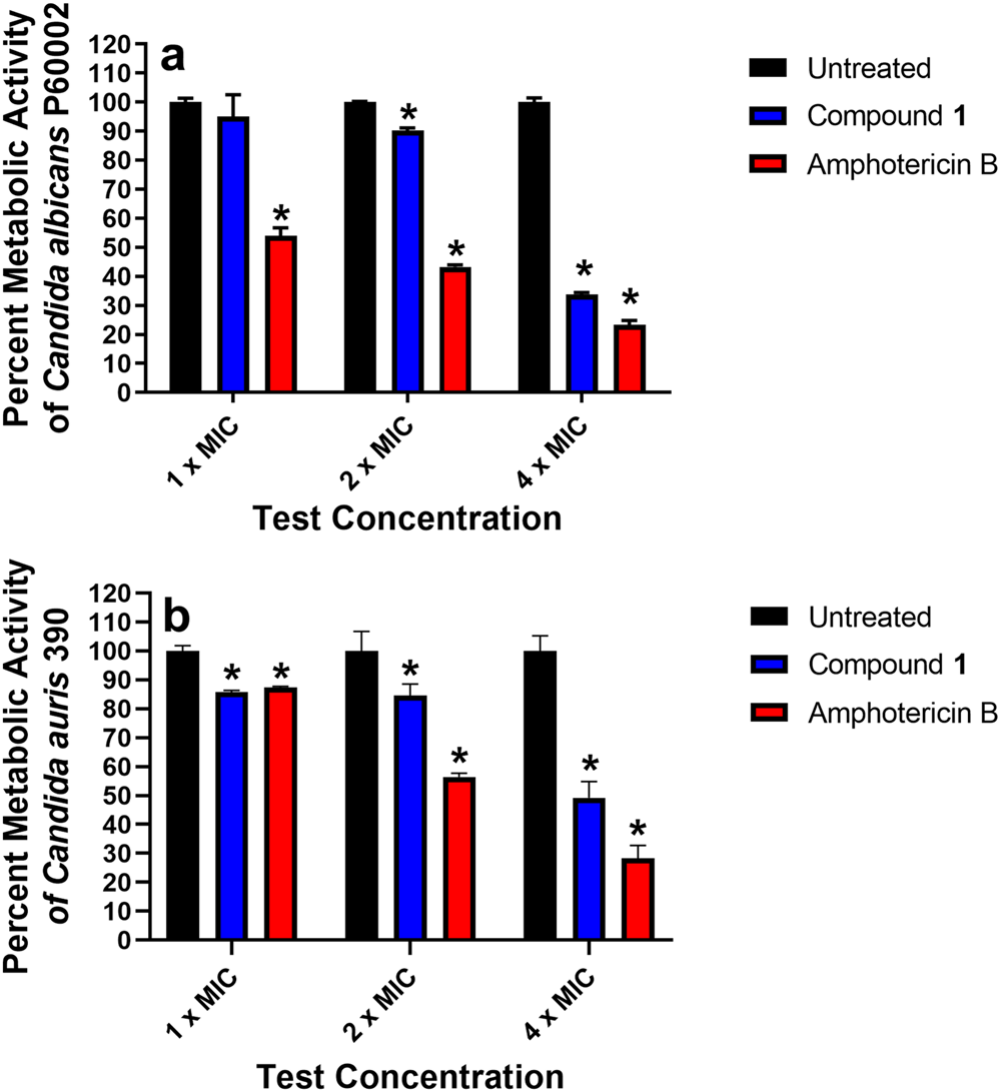
Antibiofilm activity of compound 1 and amphotericin B against mature *Candida* biofilm. Test agents were evaluated against (**a**) *C*. *albicans* P60002 and (**b**) *C*. *auris* strain 390 adherent biofilm evaluated with the XTT assay. Adherent biofilms were treated with either compound **1** or amphotericin B (both in triplicate) at the concentrations presented, over a 24-hour period. The percent metabolic activity for each treatment was calculated relative to untreated wells. Error bars represent standard deviation values. Asterisk denotes statistical difference between compound **1** and amphotericin B relative to the negative control (untreated wells) evaluated using a two-way ANOVA, with post hoc Dunnet’s test for multiple comparisons (*P* < 0.05).

The ability of compound **1** to disrupt pre-formed adherent biofilm was tested using the XTT reduction assay. Compound **1** showed concentration-dependent disruption of *Candida* biofilm metabolic activity. Against *C*. *albicans* P60002, compound **1** reduced the metabolic activity of cells present in the biofilm by 5% (at 1 × MIC), 9.7% (at 2 × MIC; *P* = 0.0023), and 66.3% (at 4 × MIC; *P* < 0.0001), relative to the untreated control (Fig. 3a). Amphotericin B was more effective at higher concentrations, where the drug (*P* < 0.0001) reduced metabolic activity of *C*. *albicans* present in the biofilm by 46.1% (at 1 × MIC), 56.8% (at 2 × MIC), and 76.6% (at 4 × MIC), relative to the untreated control. Both compound **1** and amphotericin B, at higher concentrations, had potent antibiofilm activity against *C*. *auris* strain 390. Compound **1** significantly reduced the metabolic activity of cells present in the *C*. *auris* biofilm by 14.3% (at 1 × MIC; *P* = 0.0112), 15.5% (at 2 × MIC; *P* = 0.0072), and 50.7% (at 4 × MIC; *P* < 0.0001), relative to the untreated control (Fig. 3b). Amphotericin B significantly reduced activity of *C*. *auris* cells in the biofilm by 12.7% (at 1 × MIC; *P* = 0.0212), 43.5% (at 2 × MIC; *P* < 0.0001), and 71.6% (at 4 × MIC; *P* < 0.0001), relative to untreated control.

### Safety profile of compound 1 against mammalian cells

**Figure 4:**
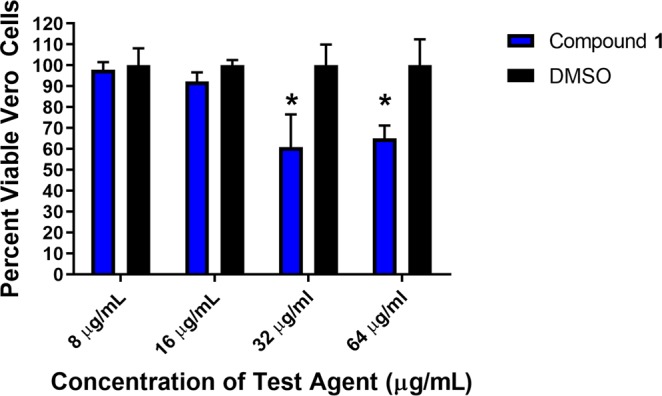
Toxicity of compound 1 against monkey kidney epithelial cells (Vero). Cells were exposed to compound **1** for 24 hours. Data represent percent viable cells after exposure to the compound (tested in triplicate) at 8, 16, 32, and 64 μg/mL using the MTS assay. Dimethyl sulfoxide (DMSO) was used as a negative control. Error bars represent standard deviation values. An asterisk (*) denotes statistical difference between compound **1** and DMSO evaluated using a two-way ANOVA, with post hoc Sidak’s multiple comparisons test (*P* < 0.05).

The toxicity of compound **1** to mammalian cells was evaluated using a kidney epithelial cell line (Vero) at concentrations ranging from 8 to 64 µg/mL. No acute toxicity was observed for Vero cells exposed to compound **1**, up to the maximum concentration tested, after two hours of exposure. When the exposure was increased to 24 hours, Vero cells appeared unaffected by compound **1** up to 16 µg/mL (Fig. 4). This concentration is seven- to 31-fold higher than the MIC values for compound **1** against all strains of *Candida* tested.

### Compound 1 is safe to nematodes and prolongs survival of nematodes infected with *Candida*

**Figure 5:**
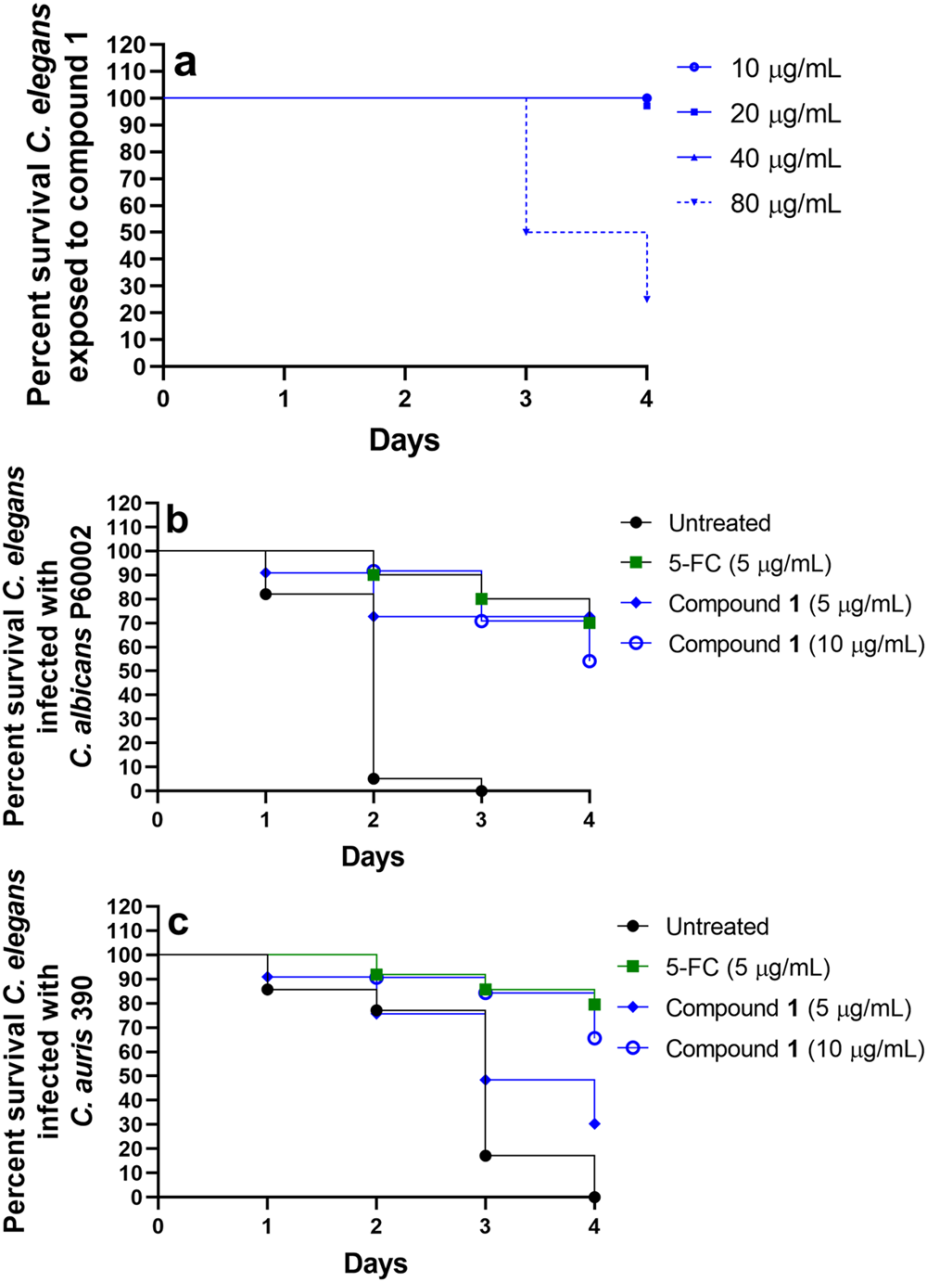
Toxicity of compound 1 and efficacy of compound 1 and 5-Fluorocytosine in *C*. *elegans* infected with *C*. *albicans* or *C*. *auris*. (**a**) Adult (L4-stage) worms were exposed to compound **1** at four different concentrations and viability was recorded daily for four days. Adult (L4-stage) worms were infected with the highly-virulent, fluconazole-resistant strains (**b**) *C*. *albicans* P60002 or (**c**) *C*. *auris* strain 390 for 90 minutes at 25 °C. Worms were washed and then treated with compound **1** (either at 5 µg/mL or 10 µg/mL), 5-Fluorocytosine (5-FC, 5 µg/mL) or left untreated. Survival of worms was monitored daily and recorded. Data are presented as a Kaplan-Meier survival curve.

After evaluating the safety profile of compound **1** to Vero cells, we evaluated the safety of compound **1** administered to *C*. *elegans*. Nematodes were exposed to 10, 20, 40, or 80 µg/mL of compound **1**, and survival was monitored daily (Fig. 5a). All worms exposed to either 10 or 40 µg/mL of compound **1** survived throughout the duration of the study (four days). In the group exposed to 20 µg/mL of compound **1**, 97% of worms survived over four days (only one worm died). A concentration of 80 µg/mL compound **1** was found to be toxic as 50% of worms died after just three days and 75% died within four days.

The efficacy of compound **1** was tested in a *C*. *elegans* model of *Candida* infection. As candidemia and disseminated candidiasis tend to occur in immunocompromised adults and children, we utilized *C*. *elegans* AU37 (sek-1; glp-4) strain (glp-4(bn2) nematodes, which are immunocompromised. Nematodes were infected with strains of *C*. *albicans* (P60002) or *C*. *auris* (strain 390) exhibiting high-level resistance to fluconazole (MIC > 64 µg/mL). Infected nematodes were subsequently treated with compound **1** (either 5 or 10 µg/mL), 5-Fluorocytosine (5 µg/mL), or left untreated and monitored daily for survival. After the second day post-infection, 94% of *C*. *albicans*-infected nematodes in the untreated group died and all nematodes were dead by the third day (Fig. 5b). In contrast, more than 70% of nematodes infected with *C*. *albicans* treated with compound **1** (both at 5 and 10 µg/mL) survived three days after infection. After four days, more than 70% of *C*. *albicans*-infected worms treated with either compound **1** (at 5 µg/mL) or 5-Fluorocytosine were alive.

Less than 20% of nematodes infected with *C*. *auris* survived three days post-infection in the untreated group, and all were dead by the fourth day (Fig. 5c). Nearly half (48%) of nematodes treated with compound **1** (5 µg/mL) survived three days post-infection and 30% of worms survived up to four days. A higher dose of compound **1** (10 µg/mL) prolonged survival of infected nematodes as nearly 70% were alive four days post-infection, similar to 5-Fluorocytosine.

## Discussion

*Candida* infections are a significant healthcare problem globally. The emergence of multidrug-resistant *C*. *auris* isolates worldwide has exacerbated this treatment challenge. The limited number of antifungal drugs combined with the development of resistance to these agents presents a challenge to treat invasive *Candida* infections. Existing antifungal drugs have a number of drawbacks including narrow antifungal spectrum, undesirable drug-drug interactions due to the inhibition or induction of cytochrome P450 enzymes (particularly for azoles), toxicity to host tissues (such as nephrotoxicity for polyenes and hepatotoxicity for 5-Fluorocytosine), and a lack of suitable oral formulations (namely for polyenes and echinocandins)^5,10,22^. Though several new antifungal agents are currently being investigated in clinical trials, the small number of antifungals with unique mechanisms of action combined with the low success rate of anti-infectives to receive regulatory approval necessitates the ongoing need to discover new antifungal agents^5,10,23^.

Thiazole compounds are often present in natural products and have been found to have potent anti-infective properties including as antibacterial, antimalarial, and antiviral agents^24^. Additionally, as discussed by Singh *et al*., more than 20 FDA-approved drugs and over 70 experimental drugs contain the thiazole scaffold in their core structure, highlighting the importance of this scaffold in drug discovery^24^. Moreover, several phenylthiazole compounds have been reported to possess antifungal activity against *C*. *albicans* and species of *Cryptococcus*^25–27^. In the present study we evaluated 85 phenylthiazole small molecules, developed in-house, for antifungal activity against fluconazole-resistant *C*. *albicans* P60002. Previously these small molecules were extensively investigated as antibacterial agents against drug-resistant staphylococci and enterococci^28–35^. Interestingly, the most potent phenylthiazole compounds with antibacterial activity were generally less effective against *C*. *albicans*. For example, compounds **2** and **3** inhibited growth of drug-resistant *S*. *aureus* isolates at a concentration as low as 0.50 µg/mL and 0.70 µg/mL, respectively^31,32^. However, both compounds inhibited growth of *C*. *albicans* P60002 at 4 µg/mL. More than 70 of the phenylthiazole compounds tested exhibited weak antifungal activity (MIC ranged between 16 to 64 µg/mL) or were inactive against *C*. *albicans* P60002. Compound **1** was selected for further investigation as it was the molecule with the strongest antifungal activity (MIC = 0.50 µg/mL), with a potency similar to amphotericin B.

A limitation with certain first-generation triazole antifungals, such as fluconazole, is their narrow spectrum of activity. Recently, increased resistance to azoles has been observed both in non-*albicans Candida* species as well as in isolates of *A*. *fumigatus*^10^. Thus, we evaluated whether compound **1** would exhibit a limited spectrum of antifungal activity similar to fluconazole. Compound **1**’s antifungal activity was assessed against a broad range of *Candida* species (including strains of *C*. *auris*, *C*. *glabrata*, *C*. *parapsilosis*, and *C*. *tropicalis*) as well as against other important pathogenic fungi (namely species of *Cryptococcus* and *Aspergillus*). Against eight *C*. *auris* isolates tested, compound **1** inhibited growth of these strains at 2 µg/mL which was superior to fluconazole (MIC > 64 µg/mL for seven isolates) and nearly equipotent to amphotericin B (MIC ranged from 0.50 to 2 µg/mL). Against *C*. *glabrata* isolates resistant to fluconazole (MIC ranged from 16 to 32 µg/mL), compound **1** (MIC ranged from 0.25 to 1 µg/mL) exhibited similar potency to amphotericin B (MIC ranged from 0.50 to 1 µg/mL). Compound **1** possessed similar activity as both fluconazole and amphotericin B against isolates of *C*. *parapsilosis* and *C*. *tropicalis* (MIC ranged between 0.50 and 1 µg/mL). Compound **1** was more potent than both amphotericin B and fluconazole against strains of *Cryptococcus neoformans* and *C*. *gattii* while compound **1** displayed similar antifungal activity to amphotericin B against strains of *Aspergillus fumigatus*. Overall, compound **1** displayed a broader spectrum of antifungal activity, compared to fluconazole, against species of *Candida*, *Cryptococcus*, and *Aspergillus*.

As invasive *Candida* infections tend to occur in immunocompromised patients, a fungicidal agent would be advantageous in reducing the burden of or completely eliminating the pathogen in infected patients^36^. Utilizing a standard time-kill assay, compound **1** (at both 2 × MIC and 4 × MIC) demonstrated rapid fungicidal activity, reducing the inoculum of both *C*. *albicans* P60002 and *C*. *auris* strain 390 below the limit of detection within 30 minutes. Amphotericin B required two hours to achieve the same effect against both strains of *Candida*. Interestingly, compound **1** was previously found to exhibit rapid bactericidal activity *in vitro* against bacteria. Against methicillin-resistant *Staphylococcus aureus* USA300, vancomycin-resistant *Enterococcus faecium* ATCC 700221, and vancomycin-resistant *Enterococcus faecalis* HM-201, compound **1** completely eliminated the bacterial inoculum within two hours^31,33^. A concern with rapidly -cidal agents is they may exert their effect through disruption of the cell membrane, which may be non-specific and lead to toxicity to host tissues. However, compound **1** was previously shown not to physically disrupt bacterial cell membranes through standard cell leakage assays and through bacterial cytological profiling^31,33^. We also did not observe an increase in the leakage of intracellular contents when *Candida* cells were exposed to a high concentration of compound **1** (Supplementary Fig. S1), indicating the compound does not physically disrupt the integrity of the fungal cell membrane.

It has been reported by the National Institutes of Health that biofilms contribute to more than 80% of all microbial infections in the United States^7^. As noted earlier, biofilms tend to form on the surfaces of medical devices and host tissues. Both *C*. *albicans* and *C*. *auris* are capable of forming biofilms although *C*. *albicans* is a more prominent biofilm producer amongst all *Candida* species^20^. *C*. *auris* tends to form thinner biofilms due to the lack of pseudohyphae which may hinder its ability to attach to certain surfaces, a necessary initial step to form robust biofilms^13^. Current approaches to inhibit *Candida* biofilm formation in medical devices such as catheters include coating the surface with antifungal agents or the use of “lock therapy” whereby a high concentration of an antifungal agent slowly diffuses through the lumen of a catheter before insertion of the catheter into a patient^20^. As compound **1** was very effective in inhibiting growth of planktonic *Candida* cells, we investigated the compound’s ability to inhibit *Candida* biofilm formation via the crystal violet reporter assay. Compound **1** was more effective at inhibiting *C*. *albicans* biofilm formation compared to inhibiting *C*. *auris* biofilm formation. At ½ × MIC, compound **1** was more effective than amphotericin B at reducing *C*. *albicans* biofilm formation by more than 93%. In contrast, compound **1** (at ½ × MIC) reduced *C*. *auris* biofilm formation by only 20.1%. Amphotericin B (at ½ × MIC) was more effective as it reduced *C*. *auris* biofilm formation by 64.6%. Both amphotericin B and compound **1** successfully inhibited *C*. *auris* biofilm formation at 1 × MIC similar to the result observed against *C*. *albicans*.

Once biofilms form, they are very difficult to eradicate in part due to enhanced resistance to antimicrobial agents which can lead to persistent recurring infections in patients. Of the three antifungal drug classes used clinically to treat *Candida* infections, azoles tend to be the least effective at disrupting *Candida* biofilms while echinocandins and lipid formulations of amphotericin B are more effective^37^. Multiple factors contribute to the reduced sensitivity of *Candida* biofilms to antifungal therapy. However, the presence of persister cells and secretion of extracellular polymeric substances (EPS) are key factors^20^. EPS plays a prominent role in decreasing diffusion of antifungal agents through the biofilm matrix and limits phagocytosis of *Candida* cells by host immune cells^20^. Finding antifungal agents capable of disrupting adherent *Candida* biofilms would thus be beneficial. Compound **1** previously was shown to be capable of significantly disrupting adherent biofilms formed by *Staphylococcus epidermidis in vitro*^38^. This motivated us to evaluate if the compound would be effective in disrupting *Candida* biofilms. Utilizing the XTT reduction assay, compound **1**, at 4 × MIC, successfully reduced the metabolic activity of cells present in both *C*. *albicans* and *C*. *auris* adherent biofilms by 66% and 50%, respectively. Amphotericin B, at the same test concentration, reduced the metabolic activity of cells present in both *C*. *albicans* and *C*. *auris* adherent biofilms by 76% and 71%, respectively. Thus, in addition to compound **1** possessing potent *in vitro* antifungal activity against planktonic cells, the compound also possesses antibiofilm activity against both *C*. *albicans* and *C*. *auris*.

The promising antifungal and antibiofilm activity of compound **1** *in vitro* encouraged us to evaluate this compound in a preliminary *in vivo* nematode model of *Candida* infection. However, prior to testing compound **1** in nematodes, we evaluated the compound’s safety profile to mammalian cells. Compound **1** was incubated with Vero (kidney epithelial) cells for either two or 24 hours before detecting viability of cells using the MTS assay. At the maximum concentration tested (64 µg/mL), compound **1** was safe to Vero cells after two hours of exposure. When the exposure time was increased to 24 hours, more than 90% of Vero cells remained viable when exposed to compound **1** (at 16 µg/mL). At 32 and 64 µg/mL, more than 60% of Vero cells remained viable after 24 hours of exposure to compound **1**. Previous reports have indicated that the IC_50_ of amphotericin B, a nephrotoxic drug, to Vero cells is 24.4 µg/mL, which suggests that compound **1** may be potentially safer to mammalian cells compared to amphotericin B^25^.

After confirming compound **1**’s safety profile to Vero cells, we moved to evaluate compound **1**’s antifungal efficacy in a *C*. *elegans* model of *Candida* infection. *C*. *elegans* is a well-established early-stage animal model for evaluating small molecules with anti-infective activity and has been used as an *in vivo* model for *Candida* infections previously^39^. An immunocompromised strain of *C*. *elegans* was infected with the highly-virulent strains of *C*. *albicans* P60002 or *C*. *auris* strain 390 and subsequently treated with compound **1** (at 5 or 10 µg/mL) or 5-Fluorocytosine (at 5 µg/mL). The concentrations used for compound **1** were safe to *C*. *elegans*, as previously this compound was reported to be non-toxic to *C*. *elegans* up to a concentration of 20 µg/mL^33^. In this study, we monitored survival of nematodes exposed to compound **1** at concentrations ranging from 10 to 80 µg/mL. All worms exposed to compound **1** at 10 or 40 µg/mL survived for four days while 75% of worms exposed to 80 µg/mL compound **1** died within four days. When nematodes were infected with *Candida*, compound **1** successfully prolonged survival of nematodes infected with either *C*. *albicans* or *C*. *auris*. Nearly 95% of untreated nematodes infected with *C*. *albicans* were dead by the second day post-infection and all remaining nematodes had died by the third day. In contrast, after four days post-infection, more than 70% of *C*. *albicans*-infected worms treated with either compound **1** (at 5 µg/mL) or the control antifungal drug 5-Fluorocytosine were alive. 5-Fluorocytosine is used clinically, often in combination with amphotericin B, for treatment of endocarditis, meningitis, and pyelonephritis caused by *Candida* species that are not susceptible to fluconazole treatment^9^. More than 80% of nematodes infected with *C*. *auris* died within three days post-infection and all were dead by the fourth day. In contrast, 30% of *C*. *auris*-infected nematodes remained alive when treated with 5 µg/mL of compound **1**, after four days post-infection. We suspect that the enhanced efficacy of compound **1**, at 5 µg/mL, to prolong survival of *C*. *elegans* infected with *C*. *albicans* compared to *C*. *auris* may be attributed to a difference in potency against *C*. *albicans* P60002 (MIC = 0.5 µg/mL) compared to *C*. *auris* strain 390 (MIC = 2 µg/mL). Indeed, when treated with a higher concentration of compound **1** (10 µg/mL), nearly 70% of worms infected with *C*. *auris* survived up to four days post-infection. Amphotericin B was not used as a positive control in the *C*. *elegans* study because *C*. *auris* strain 390 is resistant to this drug. Furthermore, in a pilot study, amphotericin B (both at 5 and 10 µg/mL) was found to be ineffective in prolonging survival of *C*. *elegans* infected with *C*. *albicans* P60002.

We attempted to determine the antifungal mechanism of action of compound **1**. Synthetic compounds containing the thiazole ring have been shown to inhibit different molecular targets/processes in fungi including leucyl-tRNA synthetase, the fungal antioxidant system through increased generation of reactive oxygen species, and interference with the fungal cell wall^27,40,41^. Previous reports have also identified an array of genes involved in *Candida* biofilm formation, including genes corresponding to proteins involved in mannan and β-1,6-glucan synthesis, two important polysaccharides that form the EPS matrix in biofilms^20,37^. In order to identify its mechanism(s) of action, compound **1** was subjected to chemogenomic profiling against more than 6,000 heterozygous diploid deletion strains of the model yeast *Saccharomyces cerevisiae*. We previously used this method to determine the antifungal mechanism of action of a synthetic compound, a molecule currently in clinical trials, and an FDA-approved drug which displayed potent activity against different *Candida* species^16,42,43^.We were unable to determine the antifungal or antibiofilm mechanism of compound **1** via chemogenomic profiling. In bacteria, compound **1** interferes with cell wall biosynthesis through dual inhibition of the undecaprenyl diphosphate phosphatase and undecaprenyl diphosphate synthase enzymes^33^. We investigated if similar enzymes might be a target of compound **1** by testing *Candida* strains that were heterozygous for homologous genes encoding these enzymes. Strains heterozygous for a gene encoding a compound target should exhibit increased growth sensitivity than the isogenic wild-type strain^42,43^. We tested heterozygous strains encoding homologous genes in the dolicol phosphate metabolism pathway including dolichyl pyrophosphate phosphatase (*CWH8*/C1_02250W_A) and two enzymes with dehydrodolichyl diphosphate synthase activity (*RER2*/C5_05330C_A and *SRT1*/C1_12270W_A)^44–46^. None of these strains exhibited increased sensitivity compared to wild-type in a minimum inhibitory concentration assay (Supplementary Table S2) and in the viability of the strains after 24 hours incubation with the compound, both in standard (RPMI-1640) and nutrient-rich (YPD) media. *CWH8* and *RER2* may be essential in *C*. *albicans* based on a stable haploid-based large-scale survey whereas these genes are not essential in *S*. *cerevisiae*^47^. The lack of strain sensitivity suggests that these enzymes may not be the direct or sole target of compound **1**. Further investigation is needed to clarify the mechanism(s) of action of compound **1**.

## Methods

### Synthesis of phenylthiazole compounds

Compounds were synthesized and characterized as described in previous reports. The synthetic schemes for compounds **1**-**23**^29,31,35,48,49^, compounds **24**-**31**^28^, and compounds **32**-**85**^30,34,50,51^ are presented in the supplementary information file. Compounds were prepared as stock 10 mg/mL or 1 mg/mL solutions in dimethyl sulfoxide (DMSO).

### Fungal strains and reagents used in this study

Clinical isolates of all fungal species (Supplementary Table S3) were obtained from the American Type Culture Collection (ATCC, Manassas, VA, USA), BEI Resources (Manassas, VA, USA), or the U.S. CDC (Atlanta, GA, USA). Vero cells (strain NR-10385) were acquired from BEI Resources. Amphotericin B (Fisher Scientific, Fair Lawn, NJ, USA), itraconazole (TCI, Ltd., Tokyo, Japan), 5-Fluorocytosine (TCI, Ltd., Tokyo, Japan), and fluconazole (Acros Organics, New Jersey, USA) were acquired from commercial vendors. Both amphotericin B and fluconazole were dissolved in DMSO to prepare stock 10 mg/mL solutions. Yeast extract peptone dextrose (YPD, Becton, Dickinson, and Company, Sparks, MD, USA), RPMI-1640 (Gibco, Grand, Island, NY, USA), 3-(N-morpholino)propanesulfonic acid (MOPS, Fisher Scientific, Fair Lawn, NJ, USA), phosphate-buffered saline (PBS, Corning, Manassas, VA, USA), minimum essential medium (MEM, Gibco, Grand Island, NY, USA), sodium pyruvate (Sigma-Aldrich, St. Louis, MO, USA), fetal bovine serum (FBS, Corning, Manassas, VA, USA), penicillin-streptomycin (Gibco, Grand Island, NY, USA), crystal violet (Acros Organics, New Jersey, USA), sodium 3′-[1-[(phenylamino)-carbony]-3,4-tetrazolium]-bis(4-methoxy-6-nitro)benzene-sulfonic acid hydrate (XTT, Sigma-Aldrich, St. Louis, MO, USA), and 96-well plates (CellTreat, Pepperell, MA, USA) were all purchased from commercial vendors.

### Determination of minimum inhibitory concentration (MIC)

The MIC of compounds and control drugs was determined as per the guidelines by the Clinical and Laboratory Standards Institute for yeasts (M27, 4^th^ Edition) and molds (M38-A2)^52,53^. Microtiter plates containing fungi and compounds/drugs were incubated at 35–37 °C for at least 24 hours for *Candida* spp. and *Aspergillus* spp. or 72 hours for *Cryptococcus* spp. The MIC was confirmed via visual inspection of growth at each test concentration.

### Time-kill assay against *Candida* species

Fungal suspensions of *C*. *albicans* P60002 and *C*. *auris* 390 cells were separately diluted to 3.63 × 10^4^ and 7.2 × 10^4^ colony-forming units per milliliter (CFU/mL), respectively. The suspensions were exposed to either compound **1** (at either 2 × MIC or 4 × MIC), itraconazole (4 × MIC), or amphotericin B (4 × MIC) (in duplicate) in RPMI-1640 medium supplemented with MOPS. At specific time points, a sample was collected, diluted in PBS, and an aliquot of each dilution was transferred to YPD agar plates. Plates were incubated at 35 °C for 24 hours before viable CFU/mL was determined.

### Investigation of compound 1’s ability to inhibit *C*. *albicans* or *C*. *auris* biofilm formation

Overnight suspensions of *C*. *albicans* P60002 or *C*. *auris* 390 were centrifuged (4000 × *g* for five minutes), washed with PBS, and adjusted to a starting inoculum of ~5.0 × 10^5^ CFU/mL in RPMI-1640 supplemented with MOPS, as described in a previous report^16^. Aliquots (150 µL) of fungal suspensions of *C*. *albicans* P60002 and *C*. *auris* 390 were separately transferred to each well of a 96-well tissue-culture treated plate. Compound **1** or amphotericin B (at least two replicates per test agent) were added and serially diluted to create a concentration gradient. The plate was incubated for 24 hours at 35 °C to permit biofilm formation, as described previously^16^. The inoculum was removed and biofilms were washed three times with PBS (to remove planktonic fungi). Biofilm mass was quantified (OD_595_) using the crystal violet reporter assay^54^. The biofilm inhibition data were analyzed via an unpaired one-way t-test (*P* < 0.05), utilizing GraphPad Prism 6.0 (GraphPad Software, La Jolla, CA).

### Inhibition of metabolism in adherent *C*. *albicans* or *C*. *auris* biofilm

Overnight suspensions of *C*. *albicans* P60002 or *C*. *auris* 390 were prepared as described above. Aliquots (150 µL) of the fungal suspensions were placed in each well of a 96-well tissue-culture treated plate and incubated for 24 hours at 35 °C to form adherent biofilm, as per a previous report^16^. The inoculum was removed and biofilms were washed three times with PBS (to remove planktonic fungi). Test agents (at least two replicates per agent) were added (in RPMI-1640 medium supplemented with MOPS), serially diluted, and incubated with the *Candida* biofilms for 24 hours at 35 °C. The viability of cells in the biofilm was determined using the XTT reduction assay^55^. Data were analyzed using a two-way ANOVA, with post hoc Dunnet’s multiple comparisons test (*P* < 0.05) (GraphPad Software, La Jolla, CA).

### Safety profile of compound 1 against Vero cells

Compound **1** was evaluated against monkey kidney epithelial cells (Vero) to determine the potential toxic effect to mammalian cells *in vitro*, as described elsewhere^16^. Cells were cultured in MEM supplemented with 10% FBS, 1 mM sodium pyruvate, and penicillin-streptomycin at 37 °C with CO_2_ (5%). Control cells received DMSO alone at a concentration equal to that in drug-treated samples. The cells were incubated with compound **1** (in triplicate) in a 96-well plate at 37 °C with CO_2_ (5%) for either two or 24 hours. The assay reagent MTS (3-(4,5-dimethylthiazol-2-yl)-5-(3-carboxymethoxyphenyl)-2-(4-sulfophenyl)-2*H*-tetrazolium) (Promega, Madison, WI, USA) was subsequently added and the plate was incubated for four hours. Absorbance readings (at OD_490_) were taken using a kinetic microplate reader (Molecular Devices, Sunnyvale, CA, USA). The quantity of viable cells after treatment with each compound was expressed as a percentage of the viability of DMSO-treated control cells (average of triplicate wells ± standard deviation). The toxicity data were analyzed via a two-way ANOVA, with post hoc Sidak’s multiple comparisons test (*P* < 0.05), utilizing GraphPad Prism 6.0 (GraphPad Software, La Jolla, CA).

### Infection of *Caenorhabditis elegans* with *C*. *albicans* or *C*. *auris* and treatment with compound 1

L4-stage *C*. *elegans* AU37 (sek-1; glp-4) strain (glp-4(bn2) were used to assess the toxicity and efficacy of compound **1** to enhance survival of worms infected with either *C*. *albicans* or *C*. *auris*, using a previously described method^16,43^. For toxicity assessment, nematodes were exposed to either 10, 20, 40, or 80 µg/mL of compound **1** and survival of nematodes was checked and recorded daily for four days. For efficacy studies, fluconazole-resistant *C*. *albicans* P60002 or *C*. *auris* strain 390 was grown to log-phase (~1 × 10^7^ CFU/mL) in YPD broth. Nematodes (20–39) were added to the broth and infected with fungi for 90 minutes at 25 °C. Worms were then harvested by centrifugation, washed at least five times with sterile PBS, and subsequently treated with compound **1** (either 5 or 10 µg/mL) or 5-Fluorocytosine (5 µg/mL). One group of worms was left untreated as a negative control. Viability of nematodes was checked and recorded daily. Data are presented as percent survival of infected *C*. *elegans* utilizing a Kaplan-Meier survival curve generated using GraphPad Prism 6.0 (GraphPad Software, La Jolla, CA).

## Supplementary information

Supplementary Information
