# Supplementary material for: Identification of a Phenylthiazole Small Molecule with Dual Antifungal and Antibiofilm Activity Against Candida albicans and Candida auris
Source: Sci Rep. 2019 Dec 12;9:18941. doi: 10.1038/s41598-019-55379-1 (PMC6908612; doi:10.1038/s41598-019-55379-1)
Supplement: Supplementary file 1 — Supplementary Information [file 41598_2019_55379_MOESM1_ESM.pdf]

Identification of a Phenylthiazole Small Molecule with Dual Antifungal and Antibiofilm Activity Against *Candida albicans* and *Candida auris*

Haroon Mohammad[1,‡], Hassan E. Eldesouky[1,‡], Tony Hazbun[2,3], Abdelrahman S. Mayhoub[4,5], and Mohamed N. Seleem[1,6*]

[1] Department of Comparative Pathobiology, College of Veterinary Medicine, Purdue University, 625 Harrison St., West Lafayette, IN 47907, USA

[2] Bindley Bioscience Center, Purdue University, 1201 W State St., West Lafayette, IN 47907, USA

[3] Department of Medicinal Chemistry and Molecular Pharmacology, College of Pharmacy, Purdue University, 575 Stadium Mall Dr., West Lafayette, IN 47907, USA

[4] Department of Pharmaceutical Organic Chemistry, College of Pharmacy, Al-Azhar University, Cairo 11884, Egypt

[5] University of Science and Technology, Nanoscience Program, Zewail City of Science and Technology, October Gardens, 6th of October, Giza 12578, Egypt

[6] Purdue Institute of Inflammation, Immunology, and Infectious Disease, 610 Purdue Mall, West Lafayette, IN 47907, USA

*Corresponding Author:

Mohamed N. Seleem
Department of Comparative Pathobiology
Purdue University College of Veterinary Medicine
625 Harrison St., West Lafayette, IN, 47907
Phone: 765-494-0763 Fax: 765-496-2627
Email: mseleem@purdue.edu

‡ These authors contributed equally.

SUPPLEMENTARY METHODS

Synthetic schemes for the preparation of compounds 1-85

Scheme 1. General procedure for synthesis of thiazoles with aminoguanidine and its cyclic analogues 1-23

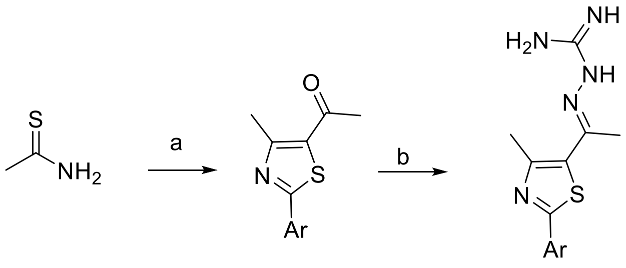

Reagents and conditions: (a) Absolute EtOH, α-chloroacetylacetone, heat at reflux (b) aminoguanidine HCl aminoguanidine HCl, or 2-hydrazinyl-4,5-dihydro-1H-imidazole HBr, EtOH, conc. HCl, heat to reflux

Scheme 2. General procedure for synthesis of Thiazolylthiazole 24-31

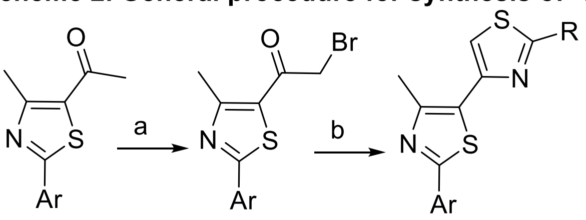

Reagents and conditions: (a) AcOH, Br_2, 65^{-75} ^oC; (b) appropriate thioamide, Absolute EtOH, K_2CO_3, heat at reflux.

Scheme 3. General procedure for synthesis of compounds 32-85

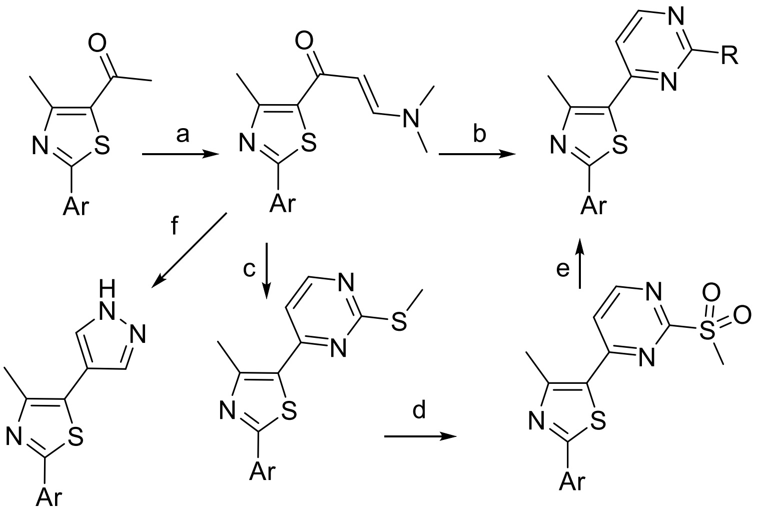

Reagents and conditions: (a) DMF-DMA heat at 80 °C; (b) proper imidines, K_2CO_3, absolute EtOH, heat at reflux; (c) i. thiourea, KOH, EtOH, heat at reflux, 8 h; ii. dimethyl sulfate, KOH, H_2O, stirring at 23 ^oC; (d) MCPBA, dry DCM, stirring at 23 ^oC; (e) appropriate amine, hydrazine, guanidine or carboximidate, dry DMF, heat at 80 ^{o}; (f) hydrazine, dry DMF, heat

***C. auris* cell leakage analysis**

An overnight culture of *C. auris* 390 cells cultured in YPD medium was centrifuged (4000 rpm for 5 minutes) and the pellet washed thrice with sterile PBS. Aliquots (1 mL) of the fungal suspension were transferred to microcentrifuge tubes and exposed to compound **1** (4 × MIC) or sodium dodecyl sulfate (2%), to completely release intracellular contents, for one hour at 35°C, similar to a previous report [1]. Untreated cells served as a negative control. Afterward, cells were centrifuged (10,000 rpm for 10 minutes), re-suspended in sterile PBS, and incubated with propidium iodide (10 µg/mL) for 15 minutes at room temperature. Propidium iodide is a membrane-impermeable dye and can only cross cellular membranes that are damaged in order to bind to DNA. The fluorescence signal was subsequently measured at an excitation wavelength of 535 nm and emission wavelength of 617 nm. Data are presented as relative fluorescent units and were analyzed via one-way ANOVA with post-hoc Dunnet’s test for multiple comparisons ($P < 0.05$).

SUPPLEMENTARY RESULTS

Supplementary Table S1: Minimum inhibitory concentration (MIC, in µg/mL) and chemical structure of phenylthiazole compounds screened against *Candida albicans* P60002.

Compound Number/Drug Name	MIC (µg/mL)	
**Thiazoles with aminoguanidine and its cyclic analogue**		
**1**	0.50	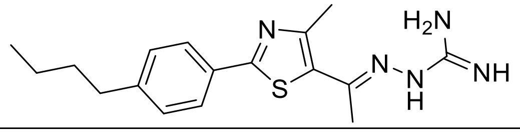
**2**	4	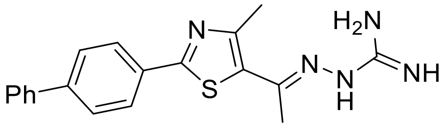
**3**	4	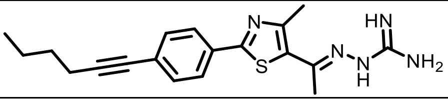
**4**	>64	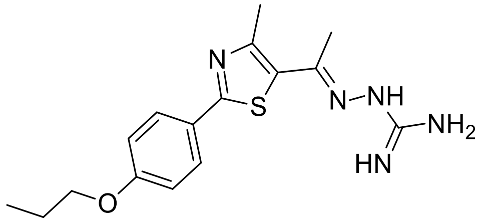
**5**	>64	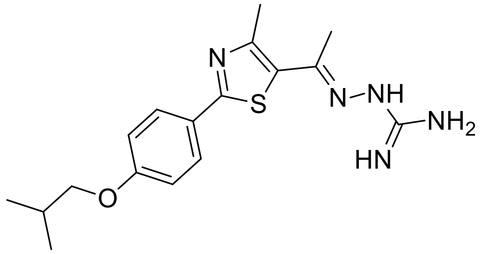
**6**	>64	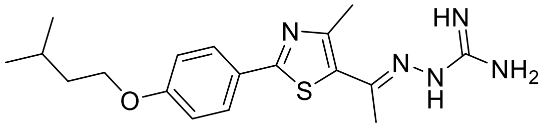
**7**	>64	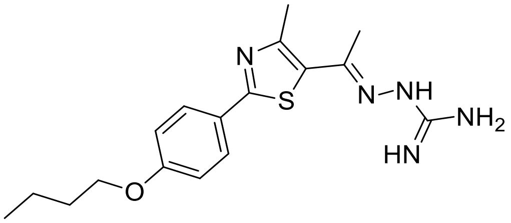

**8**	16	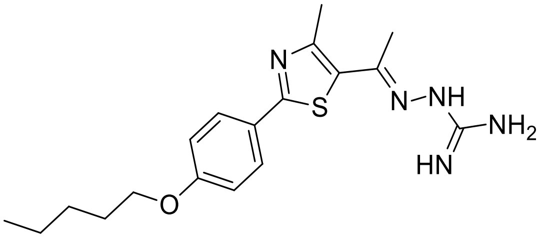
**9**	64	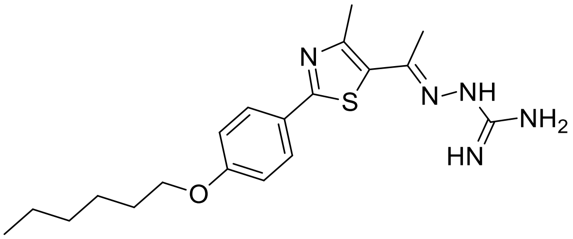
**10**	2	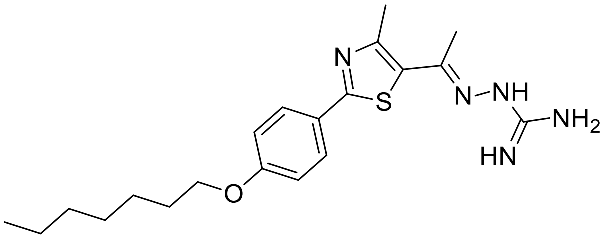
**11**	4	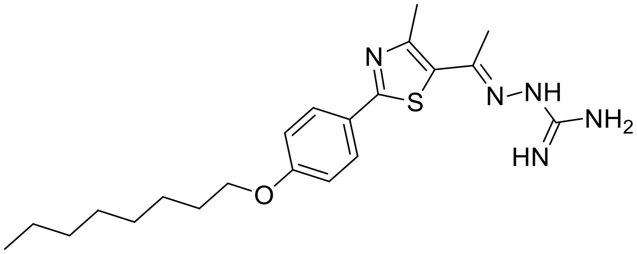
**12**	16	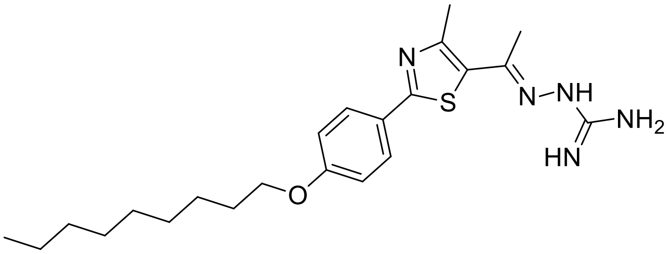
**13**	8	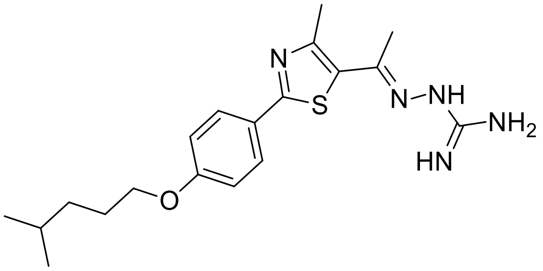

**14**	16	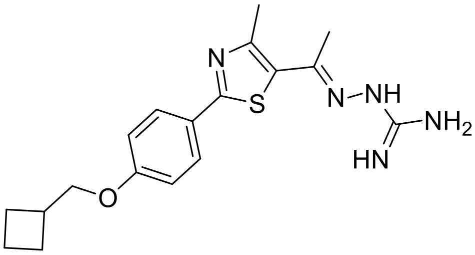
**15**	32	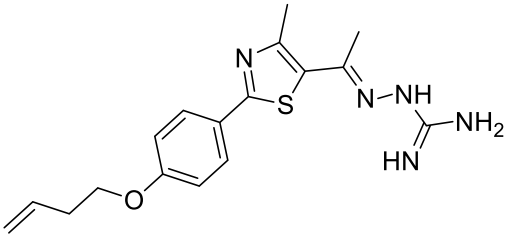
**16**	64	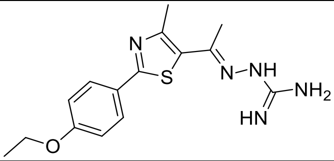
**17**	32	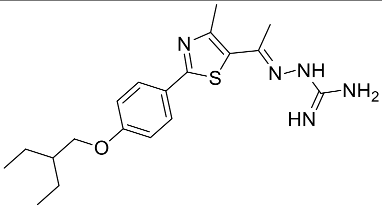
**18**	16	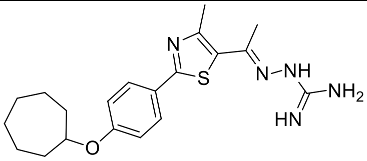
**19**	8	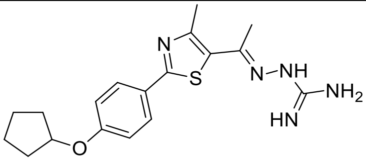
**20**	64	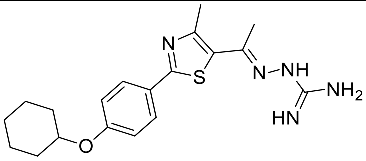
**21**	4	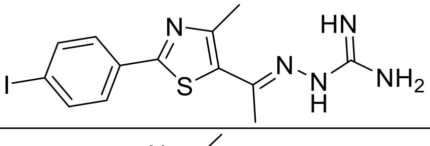
**22**	>64	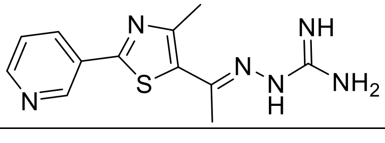

**23**	4	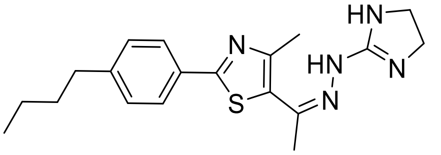
**Thiazolylthiazole analogues**		
**24**	>64	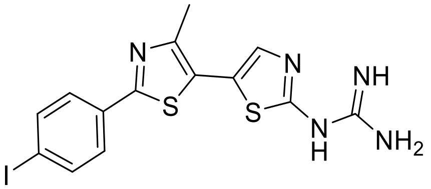
**25**	16	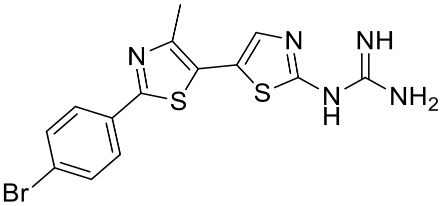
**26**	>64	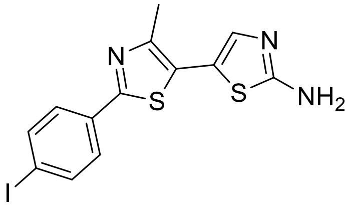
**27**	>64	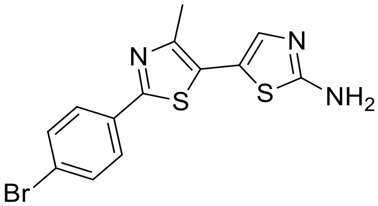
**28**	>64	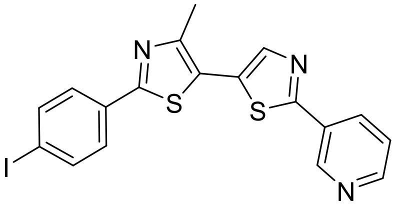
**29**	64	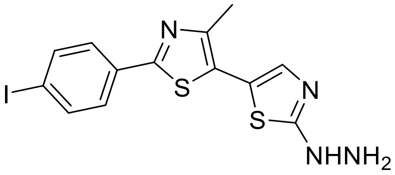
**30**	>64	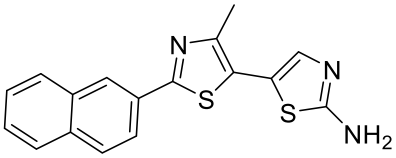

**31**	32	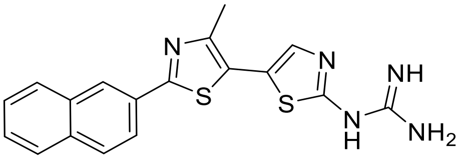
**Thiazolylpyrimidine analogues**		
**32**	>64	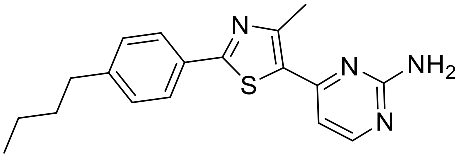
**33**	>64	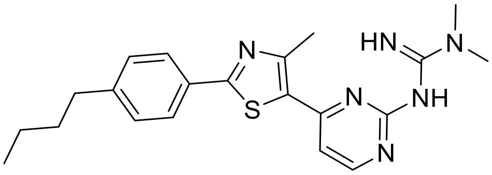
**34**	>64	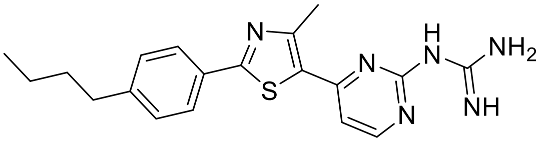
**35**	>64	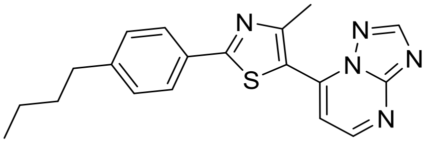
**36**	8	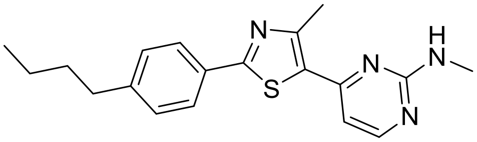
**37**	>64	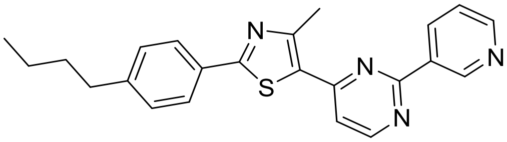
**38**	>64	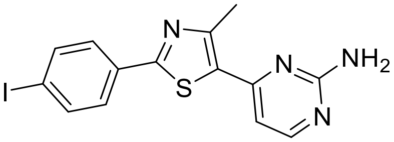
**39**	>64	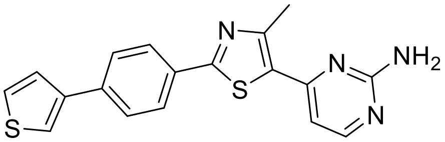
**40**	>64	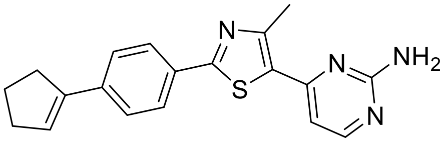
**41**	32	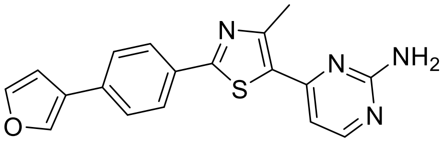

**42**	>64	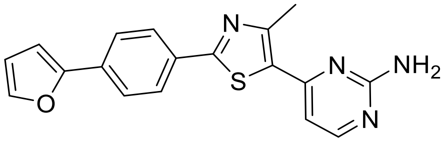
**43**	>64	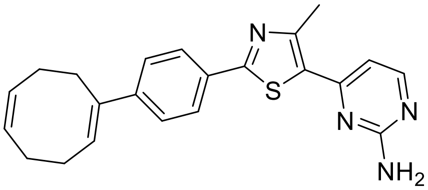
**44**	>64	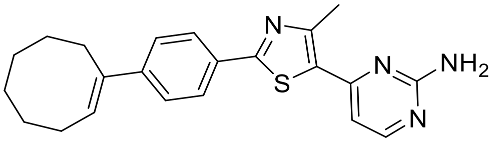
**45**	>64	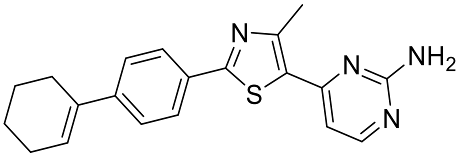
**46**	>64	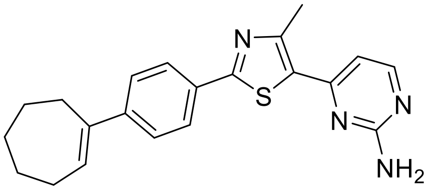
**47**	>64	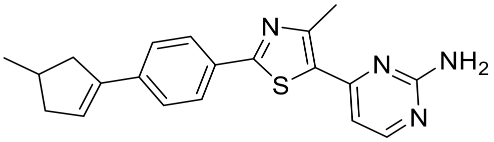
**48**	>64	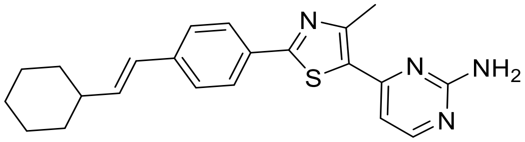
**49**	>64	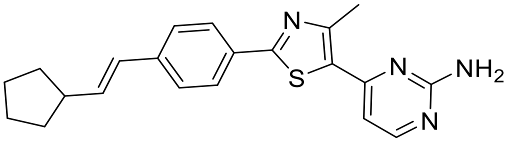
**50**	>64	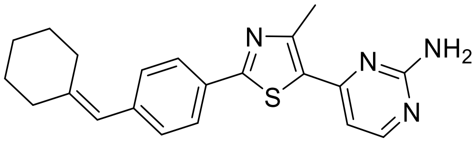
**51**	>64	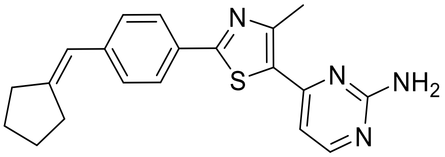

**52**	>64	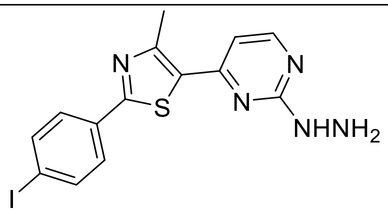
**53**	>64	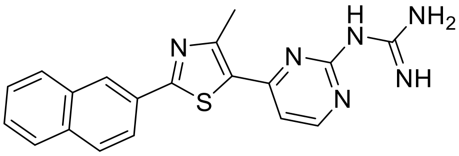
**54**	>64	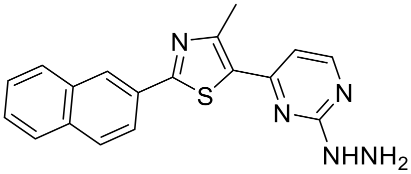
**55**	>64	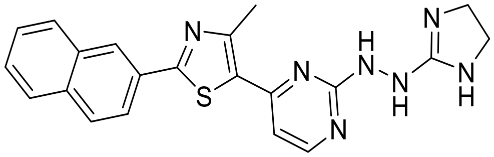
**56**	>64	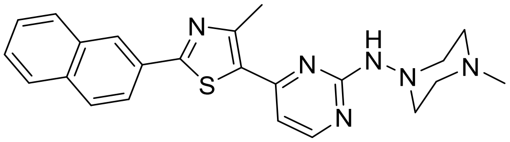
**57**	>64	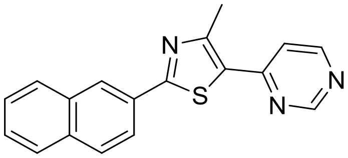
**58**	>64	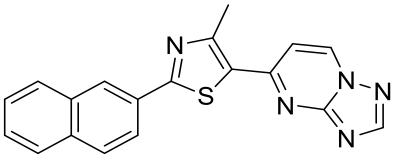
**59**	>64	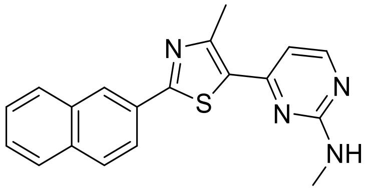
**60**	>64	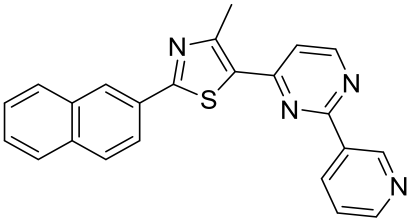

**61**	>64	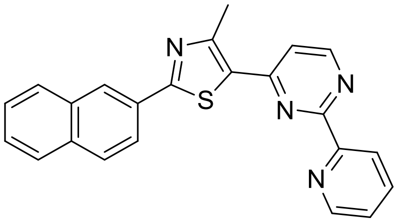
**62**	>64	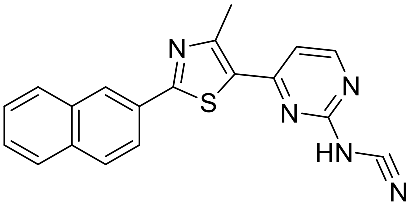
**63**	>64	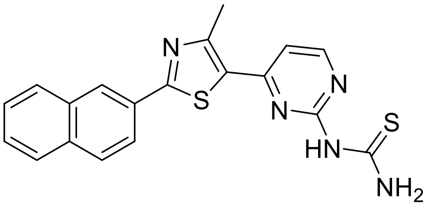
**64**	16	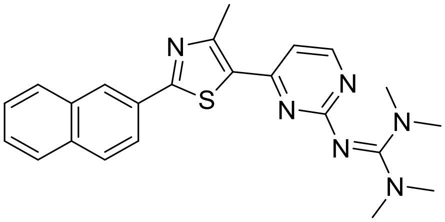
**65**	4	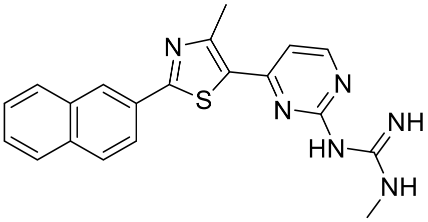
**66**	>64	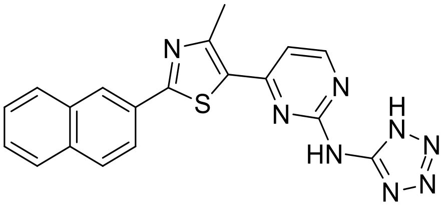
**67**	>64	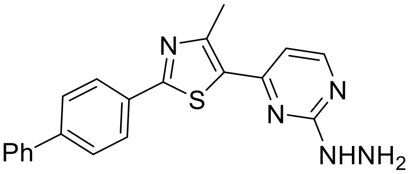
**68**	>64	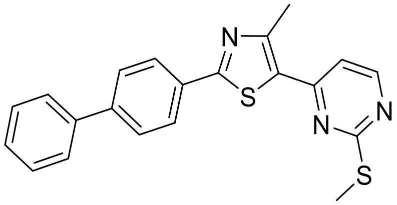

**69**	>64	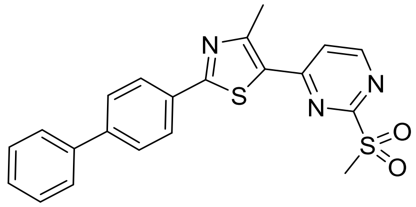
**70**	>64	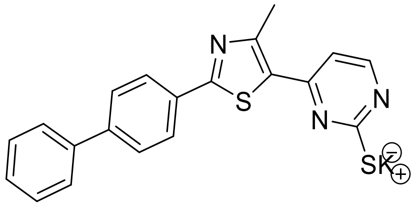
**71**	>64	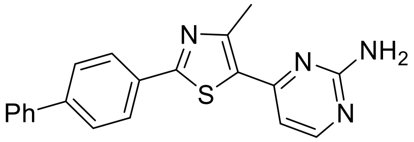
**72**	64	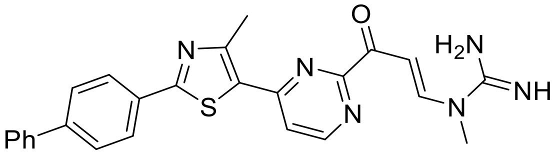
**73**	>64	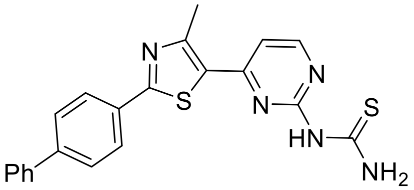
**74**	>64	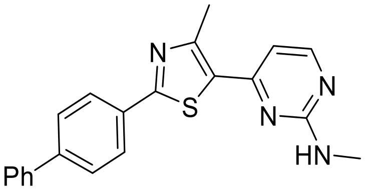
**75**	>64	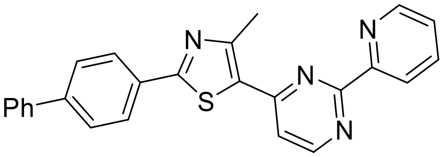
**76**	>64	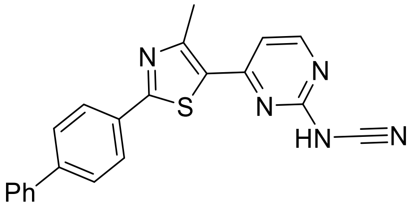
**77**	>64	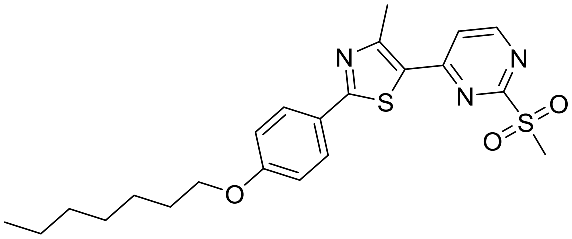

**78**	>64	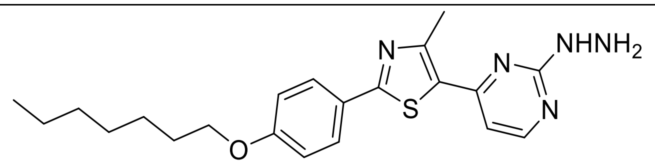
**79**	64	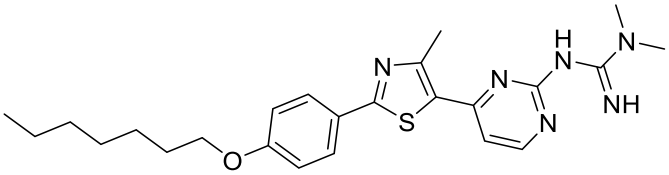
**Pyrazolylthiazole analogues**		
**80**	64	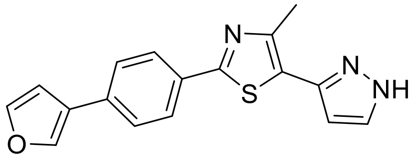
**81**	>64	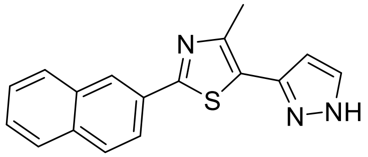
**82**	>64	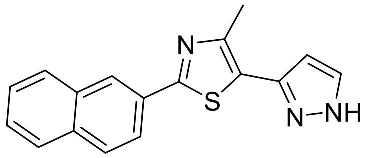
**83**	64	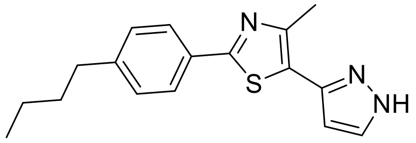
**84**	>64	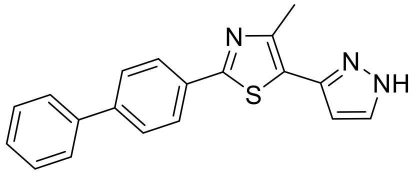
**85**	>64	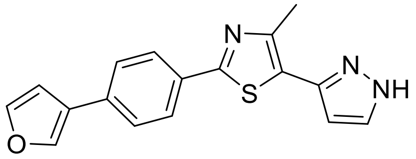
Amphotericin B	0.50	
Fluconazole	>64[1]	

[1]MIC data previously presented in reference

Supplementary Table S2: Minimum inhibitory concentration of compound **1** and amphotericin B against *C. albicans* SC5314 wild-type and three heterozygous deletion strains for genes involved in dolichol phosphate metabolism.

	**RPMI-1640 + MOPS Medium**		**YPD Broth**	
**Strain**	**Compound 1**	**Amphotericin B**	**Compound 1**	**Amphotericin B**
Wild-type	2	1	8	0.50
CWH8	2	1	8	0.50
RER2	2	1	8	0.25
SRT1	2	1	8	0.50

Supplementary Table S3: Fungal strains used in this study.

Strain Name	Alternative Strain Designation	Description
Candida albicans NR-29448	P60002	Isolated from a bloodstream infection, collected in Arizona, USA.
Candida albicans NR-29351	18M	Isolated from a patient in China.
Candida albicans NR-29365	23F	Isolated from a patient in China.
Candida albicans NR-29446	P94015	Isolated from a bloodstream infection collected in Utah, USA.
Candida albicans ATCC MYA-573	M4	Isolated from a patient with AIDS in Germany. Resistant to fluconazole.
Candida albicans ATCC 64124	Darlington	Isolated from a mouth swab. Resistant to ketoconazole.
Candida auris 381		Clinical isolate obtained from CDC
Candida auris 383		Clinical isolate obtained from CDC
Candida auris 384		Clinical isolate obtained from CDC
Candida auris 385		Clinical isolate obtained from CDC
Candida auris 386		Clinical isolate obtained from CDC
Candida auris 387		Clinical isolate obtained from CDC
Candida auris 389		Clinical isolate obtained from CDC
Candida auris 390		Clinical isolate obtained from CDC
Candida krusei ATCC 14243		None.
Candida krusei ATCC 34135	ST-112	Clinical specimen isolated from Minnesota, USA.
Candida parapsilosis ATCC 22019	CBS 604	Isolated from a case of sprue in Puerto Rico
Candida glabrata ATCC MYA-2950	303542	None.
Candida glabrata ATCC 66032	AmMS 231	None.
Candida tropicalis ATCC 1369	CCY 29-7-7	None.
Candida tropicalis ATCC 13803	FDA PCl M-59	None.
Cryptococcus gattii NR-43208	R265	Isolated from a patient on Vancouver Island, Canada in late 1990s.
Cryptococcus gattii NR-43209	CBS1930	Isolated from a goat in Aruba prior to an outbreak in Vancouver, Canada.
Cryptococcus neoformans NR-41292	Isolate 2	Isolated from human cerebrospinal fluid in China in February 2012.
Aspergillus fumigatus NR-35302		Clinical isolate obtained in 1998 from human peritoneal fluid in California, USA.

Aspergillus fumigatus NR-35304		Clinical isolate was identified in 1998 from human sputum-tracheal suction in California, USA.
Aspergillus fumigatus NR-41312		An environmental isolate was identified in 2002 (Montréal, Québec, Canada)

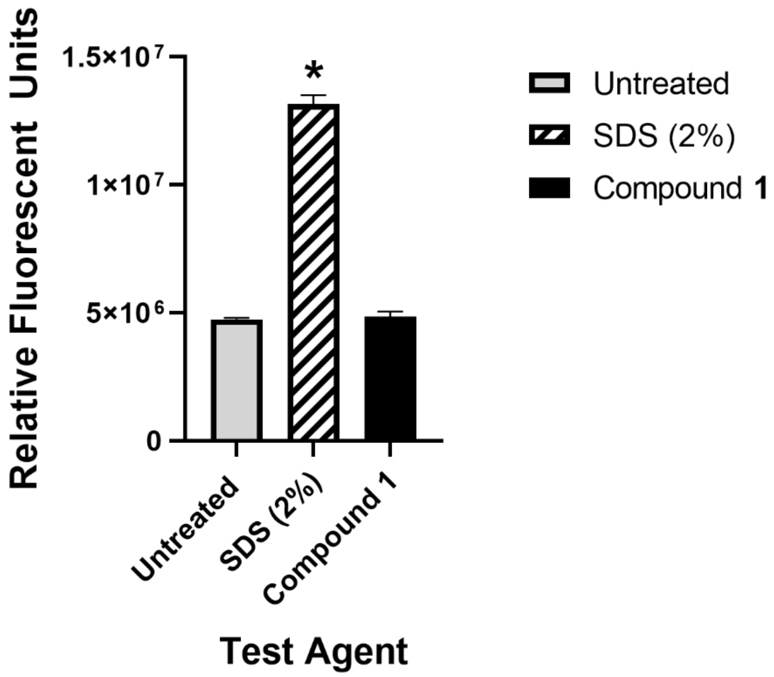

Supplementary Figure S1. Cell leakage assay for *Candida auris* strain 390 exposed to compound 1. *C. auris* 390 cells were exposed to compound **1** (4 × MIC) or sodium dodecyl sulfate (2%) for one hour at 35°C. Afterward, cells were treated with propidium iodide (10 µg/mL) for 15 minutes before recording the fluorescence signal was measured at an excitation wavelength of 535 nm and emission wavelength of 617 nm. Data were evaluated via a one-way ANOVA with post-hoc Dunnet's test for multiple comparisons ($P < 0.05$). Asterisk (*) indicates statistical difference relative to untreated cells.

REFERENCES

1 Troskie, A. M. *et al.* Synergistic activity of the tyrocidines, antimicrobial cyclodecapeptides from Bacillus aneurinolyticus, with amphotericin B and caspofungin against Candida albicans biofilms. *Antimicrob Agents Chemother* **58**, 3697-3707, doi:10.1128/AAC.02381-14 (2014).
